# An improved method for measuring catalase activity in biological samples

**DOI:** 10.1093/biomethods/bpae015

**Published:** 2024-03-05

**Authors:** Mahmoud Hussein Hadwan, Marwah Jaber Hussein, Rawa M Mohammed, Asad M Hadwan, Hawraa Saad Al-Kawaz, Saba S M Al-Obaidy, Zainab Abbas Al Talebi

**Affiliations:** Department of Chemistry, College of Science, University of Babylon, Hilla 51002, Iraq; Department of Chemistry, College of Science, University of Babylon, Hilla 51002, Iraq; Department of Medical Physics, University of Al-Mustaqbal, Hilla 51001, Iraq; Faculty of Natural Sciences, University of Tabriz, Tabriz, po 5166616471, Iran; Al-Manara College for Medical Sciences, Al-Amarah 62001, Iraq; Department of Medical Laboratories Techniques, University of Al-Mustaqbal, Hilla 51001, Iraq; Department of Chemistry, College of Science, University of Babylon, Hilla 51002, Iraq; Department of Chemistry, College of Science, University of Babylon, Hilla 51002, Iraq

**Keywords:** sulfosalicylic acid, ferrous ammonium sulfate, Bland–Altman plot, spectrophotometry, microplate protocol

## Abstract

Catalase (CAT) is an important enzyme that protects biomolecules against oxidative damage by breaking down hydrogen peroxide (H_2_O_2_) into water and oxygen. CAT is present in all aerobic microbes, animals, and plants. It is, however, absent from normal human urine but can be detected in pathological urine. CAT testing can thus help to detect such urine. This study presents a novel spectrophotometric method for determining CAT activity characterized by its simplicity, sensitivity, specificity, and rapidity. The method involves incubating enzyme-containing samples with a carefully chosen concentration of H_2_O_2_ for a specified incubation period. Subsequently, a solution containing ferrous ammonium sulfate (FAS) and sulfosalicylic acid (SSA) is added to terminate the enzyme activity. A distinctive maroon-colored ferrisulfosalicylate complex is formed. The formation of this complex is a direct result of the reaction between FAS and any residual peroxide present. This leads to the generation of ferric ions when coordinated with SSA. The complex has a maximum absorbance of 490 nm. This advanced method eliminates the need for concentrated acids to stop CAT activity, making it safer and easier to handle. A comparative analysis against the standard ferrithiocyanate method showed a correlation coefficient of 0.99, demonstrating the new method’s comparable effectiveness and reliability. In conclusion, a simple and reliable protocol for assessing CAT activity, which utilizes a cuvette or microplate, has been demonstrated in this study. This interference-free protocol can easily be used in research and clinical analysis with considerable accuracy and precision.

## Graphical Abstract

**Graphical Abstract:**
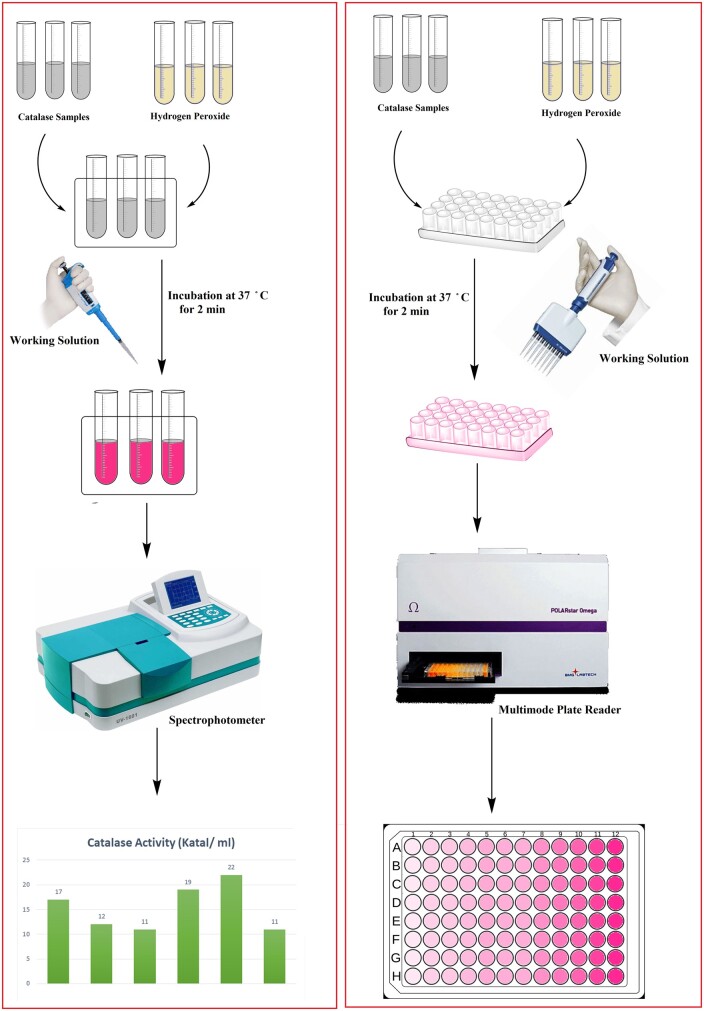

## Introduction

Oxidative stress is the main mechanism implicated in numerous pathologies and toxicities caused by xenobiotics [1]. The balance between oxidation and reduction in cells affects the signaling cascades of hydrogen peroxide (H_2_O_2_) [2]. Although H_2_O_2_ has essential signaling functions, it can also be hazardous [3]. H_2_O_2_ can be hazardous in high concentrations as it can undergo a Fenton reaction with transition metal ions, producing hydroxyl radicals (OH^•^) that cause oxidative damage to cellular components such as proteins, lipids, and DNA [4]. This can lead to changes in their structure and function, harming biological systems. The accumulation of oxidative damage can lead to chronic inflammation, accelerate aging, and play a role in the development of diseases such as cardiovascular diseases, neurodegenerative disorders, and certain cancers [5].

CAT activity is the primary mechanism for detoxifying and regulating H_2_O_2_ levels [6]. CAT is an oxidoreductase enzyme (EC1.11.1.6), part of the antioxidant enzyme group, and can be found in the cells of mammals, plants, and aerobic bacteria [6, 7]. Based on their structure and function, CATs can be classified into three main types. The first and second groups comprise heme-containing enzymes called typical or true CATs and CAT-peroxidases. The third group comprises non-heme manganese CATs [8]. The structure of CATs is a tetramer, with monomers made up of over 500 amino acids. This tetramer contains four porphyrin heme groups, which resemble those of hemoglobin, cytochromes, chlorophylls, and nitrogen-fixing enzymes [9]. CAT is present ubiquitously but is generally found in peroxisomes and has higher activity in kidneys, red blood cells (RBCs), and the liver [10]. CAT is closely related to peroxidases, both structurally and functionally, and has two functions: it reacts “peroxidatically” at lower concentrations of peroxide and “catalytically” at higher concentrations of peroxide [11].

CATs are predominately found in the peroxisome as it is the center of H_2_O_2_ production due to purine catabolism, oxidative stress, fatty acid β-oxidation, and photorespiration [12]. CATs are also found in other cellular parts like mitochondria, chloroplast, and cytosol [13]. CAT plays a crucial role in protecting HepG2 cells from ROS, and specific inhibitors that reduce CAT activity decrease their resistance to ROS. Glutathione level does not affect cellular resistance to ROS. HepG2 cells strongly resist ROS-induced apoptosis due to higher CAT activity than HeLa and other cell types [14].

CAT activity is regulated by various factors such as substrate concentration, pH, temperature, and post-translational modifications such as phosphorylation [15]. When the H_2_O_2_ concentration is high, CAT can be phosphorylated. This phosphorylation can then lead to the inhibition of the enzyme’s activity [8]. This inhibition is because phosphorylation can alter the enzyme’s conformation, making it less effective in catalyzing H_2_O_2_ decomposition [16]. However, the complete mechanism of phosphorylation inhibiting CAT activity is still not fully understood [8, 16].

Urinary tract infections (UTIs) can potentially change the level of CAT activity in urine samples. The presence of CAT activity can be used as a diagnostic indicator for certain diseases or infections [17]. However, it is important to note that relying solely on CAT activity may not be enough to establish a definitive diagnosis, and it should be used together with other clinical parameters. Normally, CAT is not found in urine [18]. However, in bacterial infections, bacteria can release CAT enzymes, which can be detected in urine samples. In UTIs, immune cells such as neutrophils can be found in urine in response to the infection [18, 19]. Also, various pathologies can result in the presence of other cells, such as epithelial cells, in urine. However, the specific types of cells found in urine can vary depending on the underlying condition, and additional diagnostic tests may be necessary for accurate identification [19].

H_2_O_2_ is a chemical compound actively produced by specialized cells, such as neutrophils [20]. These cells generate H_2_O_2_ as a defense mechanism against infections or as a byproduct of enzymatic activities, including those catalyzed by mitochondrial monoamine oxidases [21]. However, the main source of H_2_O_2_ arises from the conversion of the superoxide anion through the action of mitochondrial superoxide dismutase (SOD2) or cytosolic SOD1, which play a role in detoxification [22]. Apart from its detoxifying properties, H_2_O_2_ functions as a transcription-independent signaling molecule. It contributes to redox sensing and regulation and is as indispensable as Ca^2+^ or ATP [23]. In multicellular organisms, H_2_O_2_ regulates various transcriptional elements and thus plays a crucial role in many biological processes. CAT is another enzyme that is crucial in maintaining physiological levels of H_2_O_2,_ and plays a vital role in both preventing its cytotoxic effects and externalizing it as a threat signal [24].

Measuring CAT activity is essential for determining the redox state when assessing xenobiotic toxicity. There are several protocols available for assaying CAT activity. The first protocol involves monitoring the breakdown of H_2_O_2_ by CAT using UV spectroscopy. This method, however, requires large volumes of samples and can only measure one sample at a time [25]. The second method is titrimetric, which is suitable for tissues with low CAT levels, but this method has limitations in terms of practicality due to the large volume of samples required [2, 25]. The third method involves monitoring O_2_ generation to assess CAT activity. This method is simple, quick, and economical but is not well-suited to kinetic studies, and only one sample can be measured at a time [26].

An alternative method for measuring CAT activity involves observing the H_2_O_2_ breakdown using a suitable gel. This method requires less sample volume than the abovementioned methods, but it is worth noting that it only provides a qualitative result [27].

Chemiluminescence can be employed to assess CAT activity, utilizing H_2_O_2_-sensitive Cadmium telluride quantum dots (CdTe-QD). This method enables a rapid and sensitive determination of CAT activity but requires a luminescence reader and an intermediary step involving H_2_O_2_ and CdTe-QDs, which may impose certain limitations. Moreover, including CdTe-QDs in the assay increases the overall cost [28].

A previous study introduced a simple method to measure CAT activity using Pyrogallol Red (PGR) as a sensitive probe to measure H_2_O_2_ levels [29]. The method relied on the catalytic effects of molybdenum [7]. Spectrophotometric methods use various chemical compounds to generate colored complexes. Two examples of light absorption by chemical complexes are the carbonate cobaltate (III) ([Co(CO_3_)_3_]Co) complex, which absorbs light at 440 nm [29], and the peroxovanadate complex (NH_4_[VO(O_2_)SO_4_), which absorbs light at 452 nm [30].

A high-performance liquid chromatography assay was developed to measure human erythrocytic CAT activity in a previous study. The assay relies on glutathione analysis and employs a highly stable o-phthalaldehyde (OPA) derivative sensitive to H_2_O_2_. This method demonstrates suitability for measuring CAT activity at low concentrations, but it can be influenced by glutathione-related enzymes [31]. Maral *et al*. [32] employed a different method of assessing erythrocyte CAT activity across various species by measuring light emission from luminol oxidation catalyzed by horseradish peroxidase. The authors established a reference value of 100 for normal human blood CAT activity and expressed CAT activity in other animal species as a percentage of this reference value.

Different methods have been developed to measure the CAT activity of bacteria. One simple method is to use H_2_O_2_ to determine if CAT-positive bacteria are present. These bacteria convert H_2_O_2_ into oxygen, which produces bubbles [33]. Alternatively, methods, such as colorimetric and spectrophotometric assays, are more quantitative [34], but these can be costly and have some drawbacks, including complex procedures and the need for specialized kits [33].

Various methods have been developed for measuring CAT activity spectrophotometrically, but microplate-based methods are limited in number. The literature describes three microplate-based methods for measuring CAT activity. The first two methods are similar in principle and details [2, 34], as they depend on following the dissociation of H_2_O_2_ at a wavelength of 240 nm. Another microplate method involves assessing unreacted H_2_O_2_ in a ferrithiocyanate system, as explained by Cohen *et al*. [35]. The ferrithiocyanate method relies on unreacted peroxide oxidizing Fe(II) to Fe(III). Subsequently, a complex is formed with potassium thiocyanate, which has a peak absorbance (λmax) of 480 nm.

The current protocol describes a simple microplate assay for CAT activity based on spectrophotometric detection of unreacted H_2_O_2_. The assay incubates enzyme-containing samples with a phosphate buffer containing suitable concentrations of H_2_O_2_. After a specified incubation period, the assay introduces a mixture of sulfosalicylic acid (SSA) and ferrous ammonium sulfate (FAS) to stop the enzyme reaction. SSA binds to the ferric ions produced from the interaction of FAS and residual peroxide, creating a maroon-colored ferrisulfosalicylate complex. This complex is then measured using a spectrophotometer at 490–500 nm. The SSA-CAT assay is unaffected by different types of biomolecules and does not require strong, concentrated acids or protein precipitation to stop the enzyme reaction. It is a rapid and efficient method for measuring CAT activity.

## Procedure

### Chemicals

FAS hexahydrate [(NH_4_)_2_Fe(SO_4_)_2_(H_2_O)_6_; MWT: 392.14 g/mol, CAS number: 7783-85-9], SSA [C_7_H_6_O_6_S; MWT: 218.185 g/mol, CAS number: 97-05-2], hydrochloric acid (HCl, CAS number: 7647-01-0), glacial acetic acid (CH_3_COOH, CAS number: 64-19-7), H_2_O_2_ (30%, CAS number: 7722-84-1), monopotassium phosphate (KH_2_PO_4_ MWT: 136.09 g/mol, CAS number: 7778-77-0), sodium azide (NaN_3_, CAS number: 26628-22-8), and sodium hydroxide (NaOH, CAS number: 1310-73-2), were purchased from Thomas Baker (Chemicals) Pvt. Ltd The standard CAT was purchased from HiMedia (product code TC037; India),

### Instrument

UV–visible spectra were measured using a Shimadzu Spectrophotometer 1301A, which is equipped with 1 cm quartz cells. The study used a BioTek ELx800 UV-Vis reader to measure the 96-well plate accurately with Gene version 5 Software. All instruments and software were purchased from Aflo Company for Medical and Laboratory Equipment (Baghdad, Iraq).

### Reagents

Two solutions were prepared to create a pH 7.4, 50 mM phosphate buffer. Solution (i) is 6.81 g of KH_2_PO_4_ dissolved in 1 L of distilled water (DW), and solution (ii) is 8.90 g of Na_2_HPO_4_.2H_2_O dissolved in 1 L of DW. The two solutions were mixed in a 1:1.5 ratio to create a freshly prepared phosphate buffer. To prepare a 10 mM H_2_O_2_ solution, 0.34 mL of 30% (v/v) H_2_O_2_ was carefully diluted with the above phosphate buffer and adjusted to a final volume of 100 mL. This solution was freshly prepared and standardized daily, employing a molar extinction coefficient of 43.6 M^–1^ cm^–1^ at 240 nm. PBS-H_2_O_2_-NaN_3_ solution was prepared by dissolving 0.372 g EDTA, mM H_2_O_2_ solution, and 0.6501 g of NaN_3_ in 100 mL of PBS (pH 7.4, 50 mM). The final volume was adjusted to 100 mL with PBS. Standard potassium permanganate further standardized the diluted H_2_O_2_ solution [36].

For the preparation of FAS (10 mM), 0.4 g of FAS was dissolved in 100 mL of 7% (v/v) glacial acetic acid solution. Similarly, to prepare SSA (10 mM), 1.09 g of SSA was dissolved in 100 mL of 7% (v/v) glacial acetic acid solution. To prepare the working solution freshly, 100 mL of FAS solution and 100 mL of SSA solution were accurately measured and thoroughly mixed together. Protein concentration was measured by Bradford Protein Colorimetric Assay Kit (Cat. No.: E-BC-K168-M).

### Blood samples

Three milliliters of whole blood were collected and placed into a heparin tube to prepare erythrocyte lysates. The tube was centrifuged for 10 min at 400 × *g* to separate the plasma fractions and buffy-coat cells. The RBCs were washed thrice with 500 μL of 0.9% sodium chloride solution. After washing, 500 μL of the erythrocyte mixes were mixed with 2 mL of ice-cold double-DW. The mixture was vortexed for ten seconds and left in the dark for 15 min at 4°C. The resulting stock hemolysate was diluted further with a dilution factor 500 and resuspended in 50 mM phosphate buffer solution (PBS). Finally, the diluted hemolysate solutions were used as a source of CAT activity.

### Tissue preparation

Male albino rats and mice were obtained from the Bioscience Department, University of Babylon (Iraq) animal house for the experimental investigation. The liver tissues of the animals were surgically removed before assessing CAT activity. The liver was extensively cleaned with a 0.9% (w/v) NaCl solution to ensure the elimination of blood and other contaminants. The liver was then homogenized using a glass homogenizer in cold 1.15% (w/v) KCl. The homogenate was filtered through two layers of muslin to remove cellular debris and large particles. The resulting mixture was then diluted with 50 mM PBS at a ratio of 1:500. This diluted liver homogenate was used for subsequent CAT-activity assays.

## Procedure

### Standard methods for quantifying CAT activity

The thiocyanate method was utilized as a reference protocol [35].

### The UV-kinetic method

H_2_O_2_ was prepared in 50 mM phosphate buffer (pH 7.4) to create a final concentration of 5 mM. Next, 1000 μL of substrate solution was rapidly added to a cuvette with 25 μL of the sample. The cuvette was scanned in a spectrophotometer every 10 s for 5 min at 25°C using a wavelength of 240 nm. The CAT activity was calculated based on the rate of H_2_O_2_ decomposition, which is proportional to the reduction of absorbance at 240 nm [2, 25].

## The SSA-CAT assay

### Cuvette spectrophotometric protocol

In a water bath, 2 mL of 10 mM peroxide and 1 mL of diluted CAT sample were incubated at 37°C for 2 min. After completing the enzymatic reaction, 100 μL aliquots were transferred to a clean test tube containing 3 mL of a working solution. The test tube was vortexed and incubated at 25°C for 5 min. Finally, the absorbance was measured at 490 nm.

In a blank test tube, DW was used instead of CAT enzyme and H_2_O_2_. In a standard test tube, DW was used instead of CAT enzyme. In a control test tube, DW was used instead of a H_2_O_2_ solution.

### Microplate protocol

A 96-well plate was prepared with 100 µL of 5 mM peroxide mixed with 20 µL of CAT sample. The plate was incubated at 37°C for 5 min. Following this, 130 µL of working reagent was added to each well and mixed. The plate was then incubated for a further 5 min at 25°C. Finally, the absorbance was measured at 490 nm.

In a blank well, DW was used instead of CAT enzyme and H_2_O_2_. In a standard well, DW was used instead of CAT enzyme. In a control well, DW was used instead of H_2_O_2_ solution.

We used the microplate protocol for all practical experiments in this study. However, the microplate and cuvette protocols yielded identical results when practically compared.

### Calculation

The equation provided below was utilized to calculate the CAT activity:

$$\text{Catalase Activity of test kU = }\frac{2.303}{\text{t}}*[\text{l}og\frac{{\text{S}}^{\text{o}}}{\text{S - M}}]*\frac{{\text{V}}_{\text{t}}}{\text{Vs}}$$

t: time.

S°: absorbance of the standard tube.

S: absorbance of the test tube.

M: absorbance of control test (correction factor).

Vt: total volume (ml) of test tube.

Vs: volume of sample (ml).

During the procedure, it is necessary to eliminate interferences that arise from the presence of sugars, amino acids, proteins, and vitamins in the sample. We apply a correction factor known as the Control test to do this.

In this method, the absorbance observed in the test tube is attributed to two categories of substances: unreacted H_2_O_2_ and interferences present in the sample. However, in the control test tube, the absorbance solely arises from the interfering compounds found in the sample. By subtracting the absorbance of the control tube from that of the test tube, we can eliminate the influence of oxidizing compounds in the sample. Consequently, the remaining absorbance exclusively corresponds to unreacted H_2_O_2_.

### The interfering activity and matrix effect

The term “matrix effect” refers to the impact of other sample components on an analytical assay besides the analyte being tested [37]. For instance, in the CAT assay, the presence of glutathione peroxidase (GPx) in biological samples can potentially interfere with the results. However, a corrected CAT activity can be measured to mitigate such interference. Eliminating any matrix effect interference on CAT activity is a relatively simple process. An interfering activity test tube was incorporated into the assay design to counteract any interference caused by the GPx enzyme present in the sample being used.

The test tube’s CAT activity is the sum of the H_2_O_2_-dissociation activity of CAT activity and GPx activity. However, the interfering activity only reflects the H_2_O_2_-dissociation activity of the GPx enzyme. To ensure the accuracy of the present method, GPx activity was eliminated. This was done by subtracting the interfering H_2_O_2_-dissociation activity from the total H_2_O_2_-dissociation activity. This subtraction guarantees that the remaining H_2_O_2_-dissociation activity solely represents the precise CAT activity. Therefore, the measurement obtained is free from interfering factors.

The homogenous solution of liver tissues of the male albino rats was applied to assess precise CAT activity. The final CAT activity was adjusted to 500 katal/L using the carbonato-cobaltate complex method [29].

### Interfering H_2_O_2_-dissociation activity

An above 96-well plate was applied to assess interfering activity using PBS-H_2_O_2_-NaN_3_ solution instead of the original PBS-H_2_O_2_ solution. Sodium azide (NaN_3_) is added to inhibit the CAT enzyme and prevent its interaction with GPx activity. DW was used in a blank well instead of CAT enzyme and H_2_O_2_. In a standard well, DW was used instead of CAT enzyme. In a control well, DW was used instead of H_2_O_2_ solution.

### Precise CAT activity calculation

The precise CAT activity was calculated by applying the following equation:

$$\begin{array}{c} Precise\;CAT\;activity\;=\;Total\;H_{2}O_{2}-dissociation\;activity\\ —Interfering\;H_{2}O_{2}-dissociation\;activity \end{array}$$

Precise CAT activity: the H_2_O_2_-dissociation activity of CAT activity.

Total CAT activity: the H_2_O_2_-dissociation activity of CAT activity and GPx activity.

Interfering CAT activity: the H_2_O_2_-dissociation activity of GPx activity.

### Signal stability

A standard CAT solution (0.5 U/mL) was utilized to evaluate the stability of the maroon-colored chelate complex. The working solution was prepared with equal volumes of FAS and SSA. Absorbance measurements were taken at 490 nm at specific intervals, including 15, 30, 45, and 60 min, 5 h, one day, three days, and one week. This systematic method enabled us to monitor the long-term stability and persistence of the maroon-colored chelate complex.

### Linearity and sensitivity

This study evaluated the linearity and sensitivity using various CAT concentrations ranging from 0 to 8.0 U/mL. To prepare the standard CAT solution, 20 mg of standard powder (HiMedia, product code TC037; India) was dissolved in 100 mL phosphate buffer (50 mM, pH 7.0). The final CAT activity was adjusted to 8 U/mL using the carbonato-cobaltate complex method [29]. To assess the linearity of the method, it was compared to unreacted H_2_O_2_ using the ferrithiocyanate method [35], and the absorbance at 240 nm [34] was monitored using the UV-kinetic method. This comparison was conducted using a web-based program that estimates bias and compares analytical methods [38]. Limits of quantitation (LOQ) and detection (LOD) were estimated to determine the sensitivity of the SSA-CAT assay. These parameters are important to assess the lower limits of reliable quantification and detection of CAT activity within the assay [39].

### Selectivity, reproducibility, and accuracy

The robustness of the present CATCAT method was evaluated by conducting experiments with several types of interfering biomolecules. These biomolecules were dissolved in a phosphate buffer and divided into four flasks. The first flask contained only the buffer, while the second contained ribose, sucrose, glucose, and xylose; the third flask contained histidine, leucine, valine, and methionine; and the fourth flask contained bovine serum albumin and casein. The test-method accuracy in the presence of these biomolecules was determined by obtaining assay recovery values for each mixture. The results, summarized in Table 1, demonstrated that the CAT assay accurately measured CAT activity even in the presence of tested biomolecules. The table provides information on the correlation between the biomolecules and the observed percentage errors. The experiments used a standardized CAT activity level and involved enzymatic reactions with the biomolecule solutions.

**Table 1.** The correlation between measured the CAT activity and the incubation.

Prepared CAT enzyme activity (katal unit	3	3	3	3	3	3	3
Incubation time min)	1	2	3	4	5	6	7
The measured CAT activity (katal unit)^a^	2.7 ± 0.03	3 ± 0.03	3 ± 0.05	3 ± 0.05	3 ± 0.03	3 ± 0.09	2.9 ± 0.1

a Mean of triplicate measurement.

Biological samples from male albino rats and mice were used to assess the method’s reproducibility. The rats’ livers were surgically removed, washed, and homogenized in a cold KCl solution. The resulting liver homogenate was filtered and diluted with PBS. This diluted sample served as a source of CAT activity. The intra- and inter-day reproducibility experiments measured the variability in CAT activity within a single day and across multiple days, respectively. The results were presented in terms of the relative standard deviation (RSD).

### Validation

The ferrisulfosalicylate and ferrithiocyanate methods were compared using Bland–Altman analysis [40] and Passing–Bablok regression [41]. GraphPad Prism version 8 (GraphPad Software, San Diego, CA, USA) was utilized for statistical analysis.

## Ethics statement

### Animals

Ethics Committee (University of Babylon/College of Science/Iraq), Ref. no.: 2148A Date: 3/9/2023.

### Human

The Institutional Research Ethics Committee approved this research, and each participant completed an informed consent form. Ethics Committee (University of Babylon/College of Science), Reference number of approval: 2157A; Date: 23/12/2023.

### Statistical analysis

Data analysis was performed using GraphPad Prism version 8 statistical software (GraphPad Software, San Diego, CA, USA). The findings were reported as mean values accompanied by standard deviations. Student’s t-tests and Pearson correlations were employed to compare the studied parameters. A significance level of *P* < .05 was considered statistically significant.

## Results and discussion

**Scheme 1.**
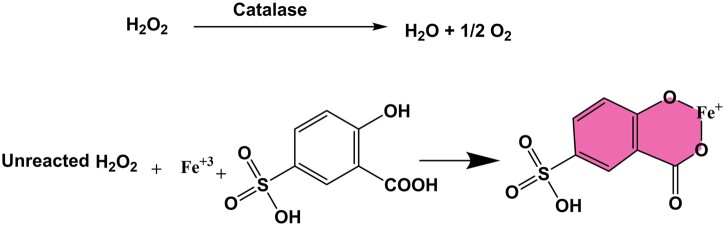
The reaction between ferric ion and SSA to form ferrisulfosalicylate complex.

Adding a reagent comprising FAS and SSA effectively terminates the CAT enzymatic reaction, as depicted in Scheme 1. After the CAT has consumed a significant portion of the H_2_O_2_, any residual H_2_O_2_ reacts with ferrous ions (Fe^2+^), leading to oxidation to ferric ions (Fe^3+^). Subsequently, salicylic acid chelates with the ferric ions, forming a complex known as ferrisulfosalicylate. This complex exhibits a distinctive maroon color, with its absorption reaching a maximum at 490 nm [42].

**Figure 1:**
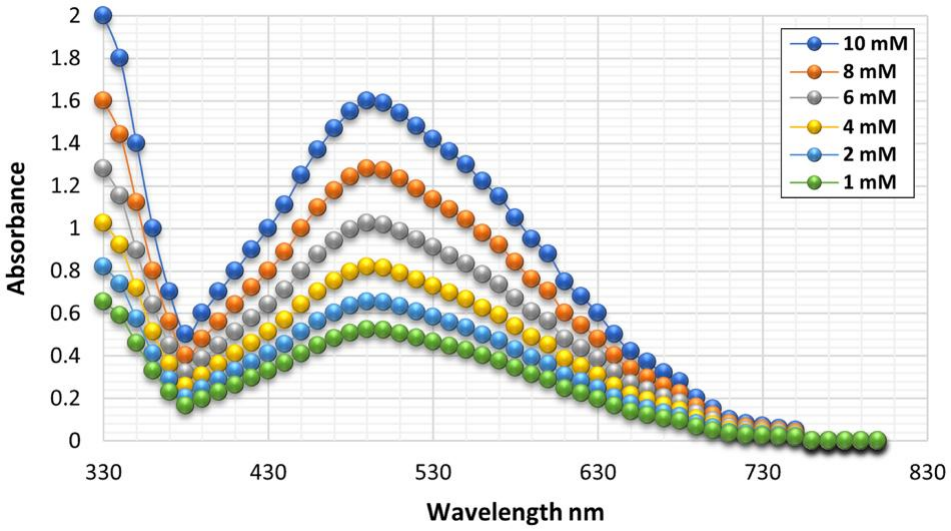
The absorbance of the ferrisulfosalicylate complex shows an inverse relationship with the activity of the CAT enzyme. The figure shows the absorption spectra of the ferrisulfosalicylate complex. The concentrations of H_2_O_2_ were (a) 10 mM H_2_O_2_ (0.54 katal unit), (b) 8 mM H_2_O_2_ (1.48 katal unit), (c) 6 mM H_2_O_2_ (2.68 katal unit), (d) 4 mM H_2_O_2_ (3.7 katal unit), (e) 2 mM H_2_O_2_ (5.01 katal unit), (f) and 1 mM H_2_O_2_ (5.84 katal unit).

Figure 1 shows a single peak at 490–500 nm, which reflects the absorbance of ferrisulfosalicylate as a function of the residual peroxide concentration from the CAT enzyme reactions. This result confirms the correlation between the absorbance values and the residual peroxide levels and supports the assay’s ability to quantify CAT activity.

**Figure 2:**
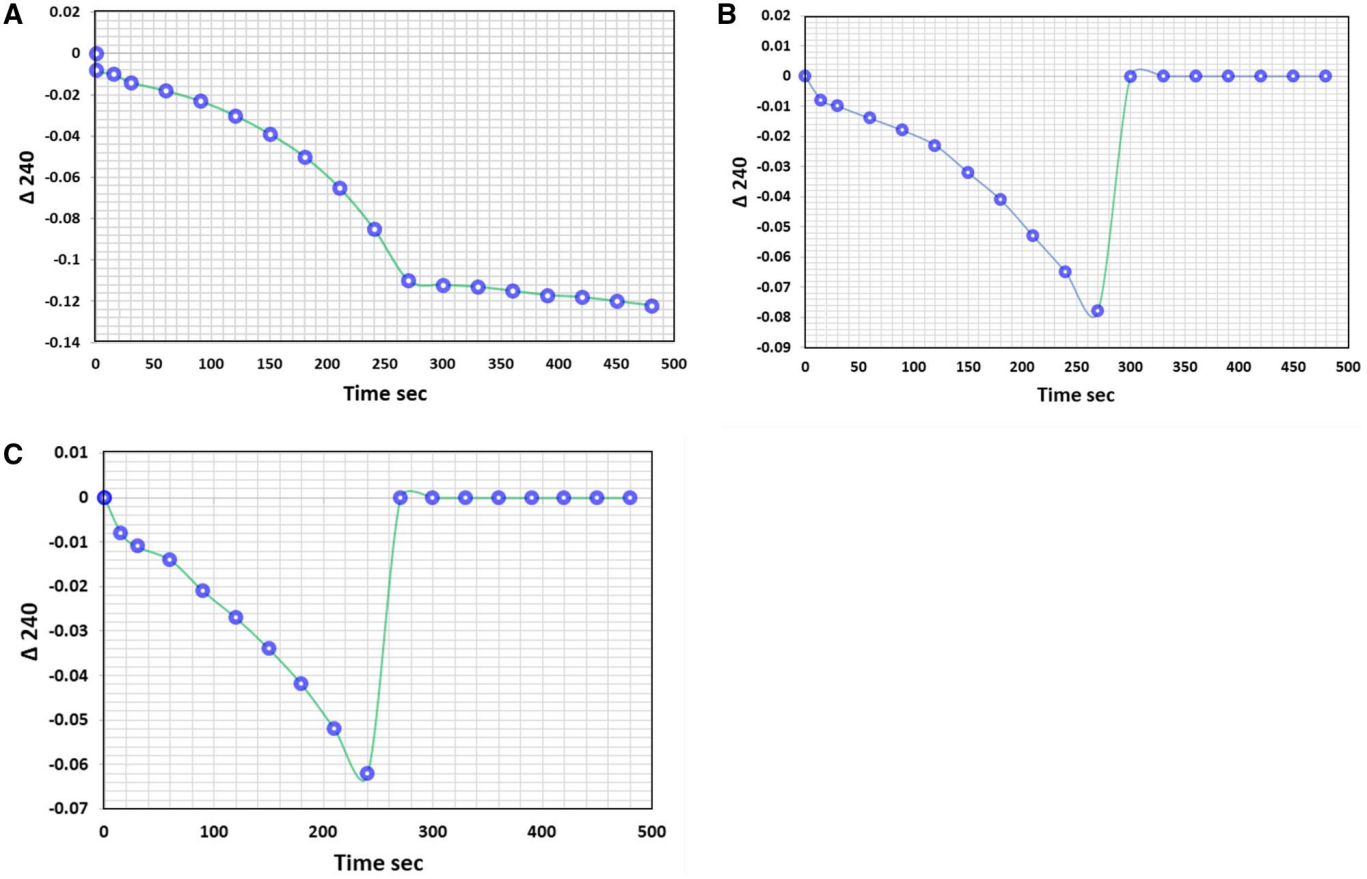
Decomposition of H_2_O_2_ over time using the CAT enzyme. The decrease in H_2_O_2_ concentration was measured by monitoring the absorbance at a wavelength of 240 nm. Three test tubes were used, each containing 3 mL of 5 mM H_2_O_2_. The reaction was initiated by adding 50 µL of 8 katal units of CAT enzyme, and the absorbance was monitored at 240 nm for 7 min. The reaction was stopped by adding 1 mL of either SSA solution (A), FAS solution (B), or working solution (C).

The effectiveness of the working solution in inhibiting the CAT enzymatic reaction was evaluated before starting the practical experiments. Three test tubes containing a standard CAT enzymatic activity of 8 U. mL^−1^ were treated with the FAS, SSA, and a combination of both (FAS/SSA) solutions. CAT activity was monitored at 240 nm. The CAT enzymatic reaction was initiated by adding freshly prepared CAT solution (8 U.mL^−1^). The results demonstrated that the FAS solution immediately stopped the CAT enzyme reaction upon addition. In comparison, the SSA solution reduced the CAT enzymatic reaction by approximately 22%. The working solution (FAS/SSA) completely inhibited the CAT reaction and exhibited similar results to the FAS solution. Figure 2 clarifies the detailed results.

This study determined the optimal incubation time for CAT through a specific experiment. The optimal incubation time ranged from 2 to 6 min, and Table 1 documents the results. These results were found to be consistent with previous studies. Li and Schellhorn [34] monitored CAT enzyme activity using 240 nm absorbance and concluded that precise results could be obtained after 1 min of incubation. Similarly, Goth [43] measured CAT activity after 1 min of incubation.

### Interfering H_2_O_2_-dissociation activity

This research assessed precise CAT activity in liver tissue homogenates. The results presented in Table 2 show precise CAT activity, total H_2_O_2_-dissociation activity, and interfering H_2_O_2_-dissociation activity.

**Table 2.** The precise CAT activity, total H_2_O_2_-dissociation activity, and interfering H_2_O_2_-dissociation activity were measured using the ferrisulfosalicylate method.

Total H_2_O_2_-dissociation activity (katal.L^−1^)		Interfering H_2_O_2_-dissociation activity (katal.L^−1^)		The precise CAT activity (katal.L^–1^)	
Mean ± SD	%	Mean ± SD	%	Mean ± SD	%
503.4 ± 3.2	100	14.4 ± 2.8	2.86	489 ± 2	97.14

Each value was expressed as the mean of five replicates.

In animals, CAT is found in peroxisomes, while GPx is found in mitochondria and cytosol. These two enzymes play complementary roles in decomposing endogenous H_2_O_2_ [44]. However, although GPx contributes to the degradation of H_2_O_2_, its interference is excluded in this study. The results presented in Table 1 indicate that the GPx enzyme interferes with the current protocol by approximately 3%. The lack of noticeable interference of GPx with CAT assessment is attributed to the outstanding catalytic efficiency of the CAT compared to the GPx enzyme.

CAT is an enzyme that has the highest turnover numbers compared to all other enzymes [45]. According to the Braunschweig Enzyme Database (BRENDA), CAT can convert over 2.8 million H_2_O_2_ molecules to water and oxygen per second using only one molecule [46]. Another study even suggests that the turnover numbers for CAT can be as high as 40 million [47]. Conversely, the BRENDA reports that the turnover numbers for GPx can range from 4.7 to 727.8 molecules per second. In brief, while GPx and CAT are essential for H_2_O_2_ dissociation, CAT stands out with its extraordinary turnover number, making it a main enzymatic powerhouse that dissociates H_2_O_2_.

The findings of this study align with the previous research conducted by Mueller *et al*. [48], which provides comprehensive insights into the decomposition of H_2_O_2_ in human erythrocytes, focusing on the roles of CAT and GPx in this process. Mueller *et al*. [48] reveal that the degradation of H_2_O_2_ by CAT exhibits a linear dependence on the concentration of H_2_O_2_. This implies that as the concentration of H_2_O_2_ increases, the activity of CAT in breaking down H_2_O_2_ also increases proportionally. This linear relationship suggests that the activity of CAT is directly influenced by the concentration of H_2_O_2_, and higher concentrations of H_2_O_2_ result in an increased rate of CAT-mediated degradation. The study establishes CAT as the main enzyme responsible for removing H_2_O_2_ in human erythrocytes, particularly at H_2_O_2_ concentrations above 10^−6 ^mol/L. In contrast, GPx becomes saturated at concentrations of H_2_O_2_ greater than 10^−6 ^mol/L. This means that at higher concentrations of H_2_O_2_, the activity of GPx in breaking down H_2_O_2_ reaches a maximum and does not further increase with additional increments in H_2_O_2_ concentration. At a concentration of 10^−6 ^mol/L, CAT exhibits a degradation rate for H_2_O_2_ that is approximately 12.5 times faster than GPx. However, when the concentration of H_2_O_2_ is increased to 10^−4 ^mol/L, the rate significantly escalates to become 100 times faster than GPx. Consequently, CAT contributes almost exclusively to the overall turnover of H_2_O_2_ at concentrations exceeding 10^−6 ^mol/L.

### Signal stability

This study observed that the colored chelate maroon complex is highly stable at room temperature. Our measurements showed that the ferrisulfosalicylate complex’s absorbance at 490 nm remained stable for over a week at 25°C. The initial absorbance was 1.6, whereas the absorbance decreased to 1.593 after a week. The data are not shown here.

### Sensitivity and linearity

**Figure 3:**
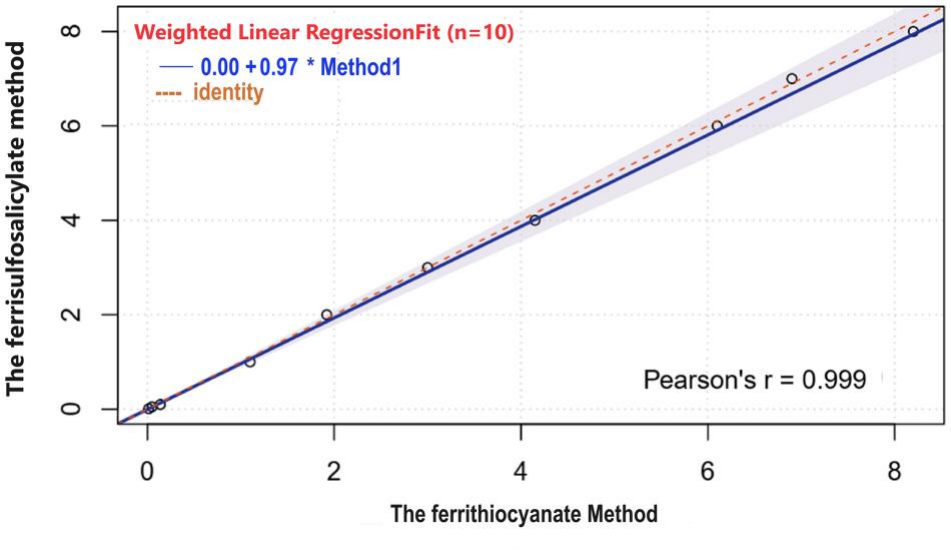
The linearity of the CAT activity method was determined by plotting a straight line between the ferrithiocyanate and ferrisulfosalicylate methods for a series of dilutions.

**Figure 4:**
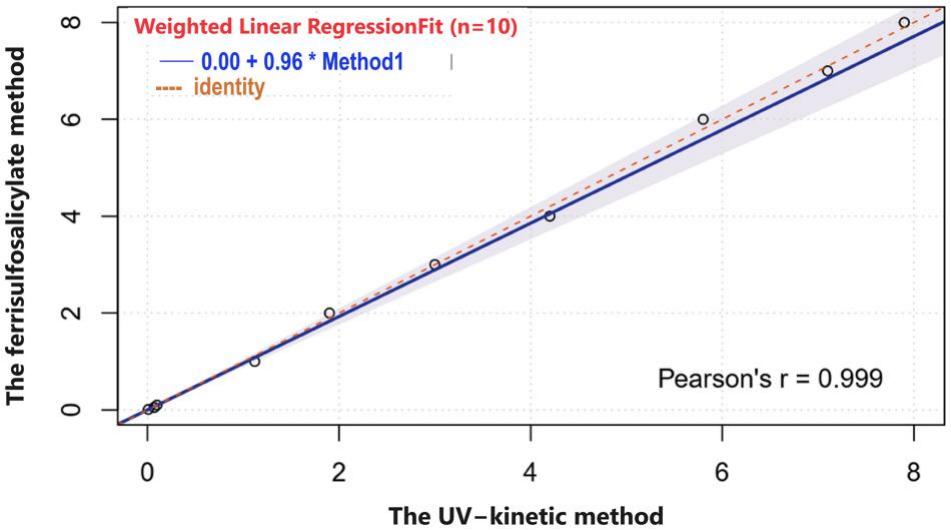
The linearity of the CAT activity method was determined by plotting a straight line between the UV-kinetic method and ferrisulfosalicylate methods for a series of dilutions.

Figure 3 shows a strong positive correlation (0.999) between the ferrithiocyanate and ferrisulfosalicylate methods. The line equation is y = 0.99x − 0.01, where y represents CAT activity measured by the ferrisulfosalicylate method and x represents CAT activity measured by the ferrithiocyanate method. In contrast, Fig. 4 compares the ferrisulfosalicylate method with the UV-kinetic method. The plot reveals a strong positive correlation (0.998) between the UV-kinetic and ferrisulfosalicylate methods. The line equation is y = 1.0072x − 0.0264, where y represents CAT activity measured by the ferrisulfosalicylate method and x represents CAT activity measured by the UV-kinetic method.

The ferrisulfosalicylate method exhibits linearity for CAT enzyme activity values ranging from 0.1 to 8.0 U/mL. The LOQ and LOD values are 0.09 U/mL and 0.022 U/mL, respectively. These low LOQ and LOD values indicate high sensitivity of the SSA-CAT assay, enabling the detection of low levels of CAT enzyme activity. The linearity of the ferrisulfosalicylate method is comparable to that of the ferrithiocyanate and UV-kinetic methods, indicating its reliability in measuring CAT enzyme activity.

### Reproducibility, selectivity, and accuracy of the SSA-CAT assay

Table 3 shows the results of an experiment examining the possibility of different biomolecules interfering with the ferrisulfosalicylate method. The lack of apparent interference indicates that the presence of these biomolecules did not significantly influence or distort CAT activity assessment when using our method, which increases the usefulness of the method.

**Table 3.** Correlation between relative percentage errors and biological interference during CAT activity assessment utilizing the ferrisulfosalicylate method.

Flasks *	Added CAT U/L	Detected CAT U/L	Relative error (%)
#flask 1	300	300	0.00
#flask 2	300	311	3.67
#flask 3	300	295	1.67
#flask 4	300	308	2.67

* Flasks 1–4 are described in detail in the materials and methods above.

This research examined CAT activity in liver tissue homogenates. The results presented in Table 4 indicate that the CAT activity assessed using the ferrisulfosalicylate method corresponded to the levels obtained with the thiocyanate method. Furthermore, the assay’s intra-day precision was satisfactory, with RSD% values ranging from 3.49% to 3.86% (Table 3). Similarly, the ferrisulfosalicylate assay’s inter-day precision assessment, which examines reproducibility across samples on various days, was considered satisfactory, with RSD% values ranging from 3.8% to 4.4% (Table 3). These data validate the assay’s accuracy and precision under different experimental conditions. The low RSD% results for intra-day and inter-day precision indicate that the ferrisulfosalicylate method is accurate and precise for assessing hepatic CAT activity in liver tissue homogenates.

**Table 4.** Comparison of CAT activities in diluted liver tissue homogenates (at a ratio of 1:500) using the ferrisulfosalicylate and thiocyanate methods.

Sample**s**	CAT activity [(katal. mL^−1^) for liver homogeneous tissues]							
	The thiocyanate method				The SSA-CAT method			
	Intra-day ± SD	RSD%	Inter-day ± SD	RSD%	Intra-day ± SD	RSD%	Inter-day ± SD	RSD%
A*	5.77 ± 0.1	1.73	5.25 ± 0.07	1.3	5.33 ± 0.09	1.69	5.15 ± 0.07	1.36
B*	5.9 ± 0.08	1.36	5.75 ± 0.08	1.39	5.8 ± 0.11	1.9	5.6 ± 0.09	1.6

* Liver tissue homogenates of male albino rats (A) and mice (B).

The CAT activity can be used to assess the liver’s ability to reduce oxidative stress. Furthermore, the oxidant/antioxidant balance has been determined by several systematic investigations that have measured CAT activity in the livers of laboratory animals [49, 50].

### Validation and method comparison

**Figure 5:**
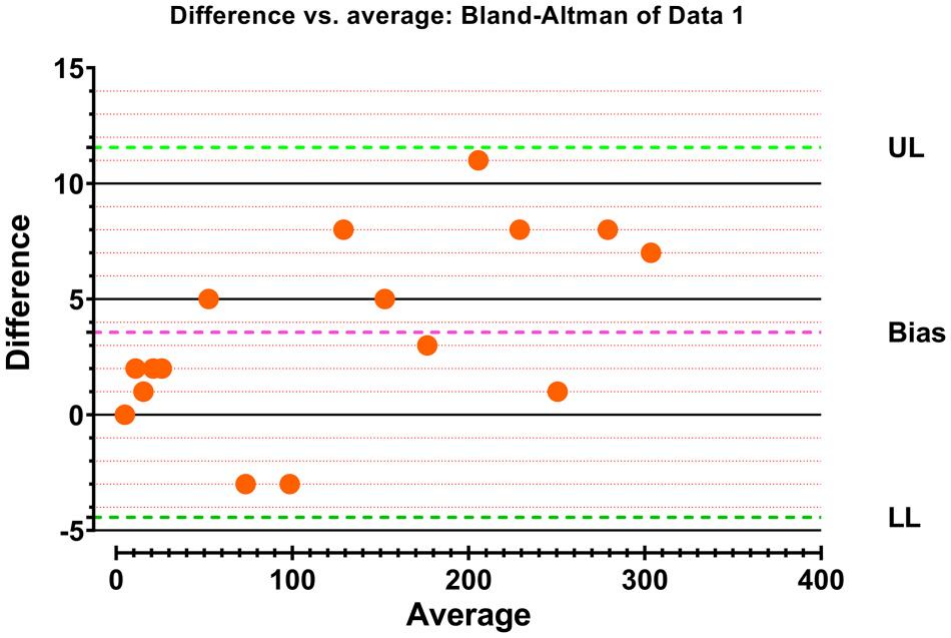
Bland–Altman plot demonstrates the differences between ferrisulfosalicylate and ferrithiocyanate methods, including their mean relative bias. %Difference = ([The ferrithiocyanate method (katal unit)−The ferrisulfosalicylate method (katal unit)]/average) × 100; average =  [(The ferrithiocyanate method (katal unit) + The ferrisulfosalicylate method (katal unit)]/2.

**Figure 6:**
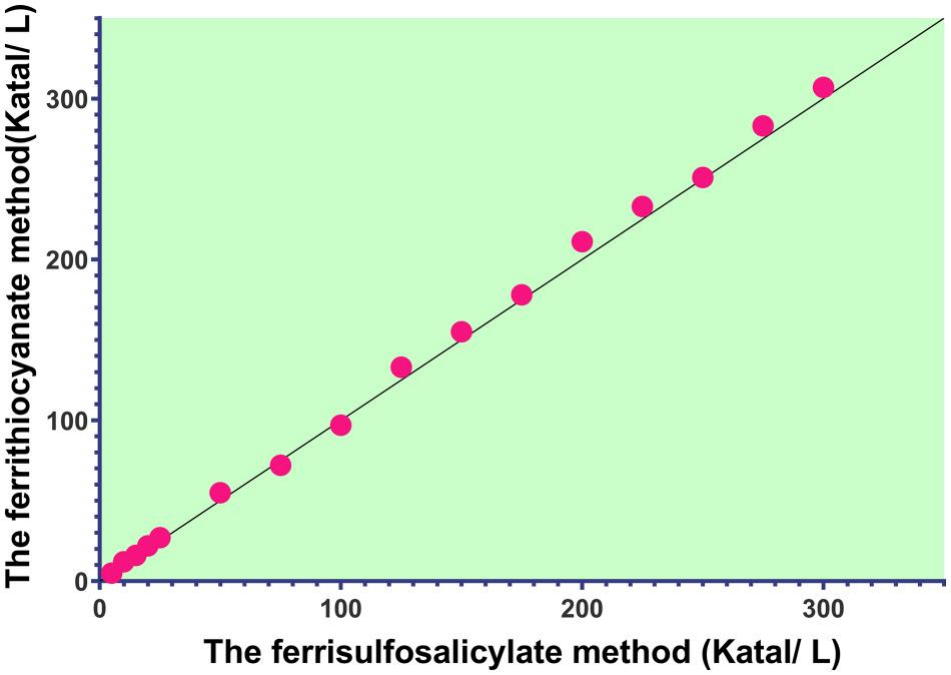
CAT activity results were determined utilizing the ferrisulfosalicylate and ferrithiocyanate methods at various enzyme dilutions.

The effectiveness of this assay for measuring CAT activities was verified by conducting Bland–Altman plot analyses using GraphPad Software in San Diego, CA, USA. To compare CAT activities, this study used ferrisulfosalicylate and ferrithiocyanate assays with paired enzymatic samples. The Bland–Altman plot in Fig. 5 shows the differences and the mean relative bias between the two methods. The correlation coefficient of 0.9968 between the ferrisulfosalicylate and the ferrithiocyanate methods confirms that this assay is as accurate as the reference method, as shown in Fig. 6. The Passing–Bablok correlation analysis demonstrates a good agreement between the two methods, as shown in Fig. 5. Pearson correlation also proved the correlation, with a Pearson r of more than 0.99 between the ferrisulfosalicylate method and the ferrithiocyanate method’s results for different samples.

### Application I

This study also conducted experiments to assess CAT activity in lysates derived from five distinct bacterial laboratory strains. The primary aim was to explore further potential applications of the SSA-CAT method. The results revealed that the ferrisulfosalicylate method yielded CAT enzyme activities comparable to those obtained through the thiocyanate method across the bacterial strains. Our findings indicated that *Staphylococcus aureus* exhibited a noticeably higher CAT enzyme activity than other bacterial strains. For in-depth information and specific data, please refer to Table 5.

**Table 5.** Comparison of the SSA-CAT and thiocyanate methods for bacterial CAT activities (katal unit).

Name of bacteria	The ferrithiocyanate method	The ferrisulfosalicylate method
*Staphylococcus aureus*	14.7	14.9
*Pseudomonas aeruginosa*	11.5	11.7
*Escherichia coli*	8.1	7.7
*Klebsiella pneumonia*	13.3	13.8
*Enterococcus faecalis*	0	0

### Application II

Analysis was performed on 100 urine samples obtained from patients who visited Prof. Dr Abdul Razzaq Alsalman’s private medical, Infertility, and Urology Clinic in Babylon Governorate, Hilla City, Iraq, between December 2023 and January 2024. The participants underwent a physical examination and provided a complete medical history. The Institutional Research Ethics Committee approved the study, and all participants signed an informed consent form.

Midstream urine samples were collected in sterilized, airtight plastic containers labeled with participant-specific codes. The samples were stored in a cold box during transportation to the laboratory. In the laboratory, the samples were subjected to urine analysis and urine culture using the method described by Berger et *al*.[51]. CAT activity was measured using the SSA-CAT assay.

Out of the 100 samples, 38 were positive for colony count and CAT determination (group I), indicating the presence of bacteria. Additionally, 57 samples that were negative for colony count also tested negative for CAT determination (group II). It was observed that the presence of RBCs in 5 urine samples led to a false positive result in CAT activity (group III) [46]. In conclusion, the SSA-CAT method proved to be a successful screening test for significant bacteriuria in CAT determinations. Table 6 shows detailed information and the CAT activity.

**Table 6.** The urine CAT activities (katal unit) were obtained using the SSA-CAT method.

Groups	*n.*	Range of CAT activity (katal unit)	Mean of CAT Activity (katal unit)
Group I	38		Not detected
Group II	57	0.3–6.6	2.5 ± 1.95
Group III*	5	2.2–23	14.85 ± 6.45

* The samples were diluted ten times with PBS (pH 7.4, 50 mM).

### Application III

The study comprised 100 male students from the College of Science at the University of Babylon, Iraq. The participants had an average age of 22.0 ± 2 years and a body mass index of 22.86 ± 1.2 kg/m^2^. Informed written consent was obtained from all volunteers after providing them with a clear explanation of the study’s purpose. The participants were then categorized into two groups: smokers and controls. The control group consisted of individuals with no smoking history, while the smokers had been smoking an average of 20 ± 5 cigarettes per day for over 2 years. All participants were non-alcoholics and were not afflicted with any chronic diseases. The institutional ethics committee approved the study before its initiation.

After an overnight fast, 5 mL of venous blood containing heparin was drawn. The blood was centrifuged at 3000 rpm for 10 min to separate the plasma from the erythrocytes. To obtain packed erythrocytes, the erythrocytes were washed multiple times with a 0.9% NaCl solution until a colorless supernatant was observed. To obtain erythrocyte hemolysate, 500 µL of packed erythrocytes were lysed by adding four volumes of cold redistilled water. The resulting mixture was centrifuged twice to remove all cell membranes: first, it was centrifuged for 10 min in a tube centrifuge at 3500 rpm at 4°C and then in an Eppendorf centrifuge at 7800 rpm for 5 min at 4°C [52]. The resulting clear supernatant was obtained as hemolysate for determining CAT activity.

According to Table 7, the CAT activity in erythrocytes is significantly lower in smokers than in nonsmokers (*P* < .05). This finding suggests that tobacco smoking is associated with a reduction in CAT activity within erythrocytes. Smoking introduces harmful substances into the body, such as reactive oxygen species (ROS) and free radicals. These substances can induce oxidative stress, overpowering the body’s antioxidant defense systems, including CAT. Continuous exposure to tobacco smoke can disrupt the balance between ROS production and CAT’s ability to neutralize them, resulting in a decrease in CAT activity. These results align with previous studies [53, 54] that have reported similar findings.

**Table 7.** Comparison of erythrocyte CAT activity of tobacco smokers and non-smokers.

	Smokers	Non-smokers	*P* value
CAT activity (katal/g. Hb)	352 ± 71	278 ± 43	<0.05

## Limitations

This study has limitations, such as the lack of kinetic information about the studied enzyme, including its kinetic parameters, enzyme-substrate binding affinity, and turnover numbers. These parameters help us understand the enzyme’s behavior and predict the effects of experimental conditions or modulators on the reaction. However, previous studies have provided all the necessary kinetic information about the CAT enzyme. The proposed method can be used to gain more insights into enzyme behavior and predict the effects of experimental conditions on the CAT enzymatic reaction.

## Conclusion

The above protocol details a method for fast and precise measurement of CAT activity. This technique is not limited to microorganisms but can also be potentially used to estimate CAT activity in animal tissues, animal fluids, and plant tissues. Therefore, it is a versatile tool. The protocol involves the use of a microplate reader. The chemicals used in this method are more environmentally friendly than those used in the past, particularly the ferrithiocyanate method, which has high toxicity and environmental risks. By replacing thiocyanate with SSA, this method aligns with the principles of green chemistry.

## Data Availability

The authors declare that all data supporting the findings of this study can be found within the article. Additional data supporting the findings of this study are available from the corresponding author upon request.
