# Correction to: An improved method for measuring catalase activity in biological samples

**DOI:** 10.1093/biomethods/bpae025

**Published:** 2024-04-26

This is a correction to: Mahmoud Hussein Hadwan, Marwah Jaber Hussein, Rawa M Mohammed, Asad M Hadwan, Hawraa Saad Al-Kawaz, Saba S M Al-Obaidy, Zainab Abbas Al Talebi, An improved method for measuring catalase activity in biological samples, *Biology Methods and Protocols*, Volume 9, Issue 1, 2024, bpae015, https://doi.org/10.1093/biomethods/bpae015

In the originally published version of this article, there was a typographical error in affiliation 4.

The affiliation has been corrected to read “Al-Manara College for Medical Sciences, Al-Amarah 62001, Iraq”.

